# CXCR2 Levels Correlate with Immune Infiltration and a Better Prognosis of Triple-Negative Breast Cancers

**DOI:** 10.3390/cancers13102328

**Published:** 2021-05-12

**Authors:** Florence Boissière-Michot, William Jacot, Océane Massol, Caroline Mollevi, Gwendal Lazennec

**Affiliations:** 1 Institut Régional du Cancer de Montpellier (ICM), Val d’Aurelle, 34298 Montpellier, France; Florence.Boissiere@icm.unicancer.fr (F.B.-M.); William.Jacot@icm.unicancer.fr (W.J.); Oceane.Massol@icm.unicancer.fr (O.M.); Caroline.Mollevi@icm.unicancer.fr (C.M.); 2 Montpellier University, 34090 Montpellier, France; 3 Institut de Recherche en Cancérologie de Montpellier (IRCM), Inserm U1194, 34298 Montpellier, France; 4 Institut Desbrest d’Epidémiologie et de Santé Publique, UMR Inserm—Université de Montpellier, 34090 Montpellier, France; 5 CNRS, SYS2DIAG, ALCEDIAG, Cap Delta, 1682 Rue de la Valsière, 34184 Montpellier, France; 6 CNRS, GDR 3697 “Microenvironment of Tumor Niches”, Micronit, France

**Keywords:** breast cancer, triple-negative breast cancer, CXCR2, chemokines, cytokines, chemokine receptors, neutrophils, checkpoint markers

## Abstract

**Simple Summary:**

Tumor microenvironment is critical for cancer progression. The role of the chemokine receptors in breast cancers is still under investigation. The aim of this study was to focus on a retrospective cohort of triple-negative breast cancers (TNBCs) and analyze the involvement of CXCR2 and its link with immune infiltration and immune checkpoint markers. High densities of CXCR2-positive cells were associated with high-grade tumors. Higher quantities of CXCR2-positive cells were correlated with elevated density of tumor-infiltrating lymphocytes (TILs), CD8+ cytotoxic lymphocytes, expression of PD-L1 by tumor and stromal cells and of PD-1 by stromal cells. In univariate analysis, low levels of CXCR2 were correlated with poor OS and RFS. In multivariate analysis, low levels of CXCR2 were associated with poor OS. Overall, our data highlight the potential beneficial association of high levels of CXCR2 with a subgroup of TNBC patients characterized by a better prognosis.

**Abstract:**

Chemokines and their receptors are key players in breast cancer progression and outcome. Previous studies have shown that the chemokine receptor CXCR2 was expressed at higher levels by cells of the tumor microenvironment in triple-negative breast cancers (TNBCs). The aim of this study was to focus our attention on a retrospective cohort of 290 TNBC cases and analyze the involvement of CXCR2, CD11b (a marker of granulocytes) and CD66b (a marker of neutrophils) and their link with immune infiltration and immune checkpoint markers. We report that high densities of CXCR2-, CD11b- and CD66b-positive cells were associated with high-grade tumors. Moreover, molecular apocrine TNBCs, defined here as tumors that express both AR and FOXA1 biomarkers, exhibited low levels of CXCR2 and CD11b. High CXCR2 and CD11b levels were correlated with elevated density of tumor-infiltrating lymphocytes (TILs), CD8+ cytotoxic lymphocytes, expression of PD-L1 by tumor and stromal cells and of PD-1 by stromal cells. On the other hand, CD66b levels were associated only with CD8+, stromal PD-L1 and PD-1 expression. In univariate analysis, low levels of CXCR2 were correlated with poor OS and RFS. In multivariate analysis, low levels of CXCR2 were associated with poor OS. Finally, in TNBC treated with adjuvant chemotherapy, CXCR2 density was associated with longer RFS. Overall, our data highlight the potential beneficial association of high levels of CXCR2 with a subgroup of TNBC patients characterized by a better prognosis.

## 1. Introduction

Four main subtypes of breast cancer have been identified, corresponding to triple-negative breast cancers (TNBCs, negative for estrogen receptor (ER), progesterone receptor (PR) and Her2), Her2-positive breast cancers and two types of luminal breast cancers (luminal A and B, positive for ER) [1]. TNBCs, which represent about 15% of all breast cancers, are characterized by poor clinical outcomes, with shorter relapse-free survival (RFS), overall survival (OS) and higher metastasis rate [1,2,3]. TNBCs have been further subclassified into different groups based on definitions that might differ between studies [4,5,6,7]. Even if the classification of TNBCs is still subject of debate, a consensus would be a division into six subtypes displaying unique gene expression profiles, including 2 basal-like (BL1 and BL2), an immunomodulatory (IM), a mesenchymal (M), a mesenchymal stem-like (MSL), and a luminal androgen receptor (LAR) subtype [5,8].

The tumor microenvironment (TME) is now considered as a key component of the tumor and includes a variety of cells (in particular B and T lymphocytes, neutrophils, macrophages, natural killer (NK) cells, dendritic cells, endothelial cells, cancer associated fibroblasts (CAFs)) but also extracellular components (cytokines, chemokines, hormones, growth factors, extracellular matrix) that surround and interact with tumor cells [9]. Tumor microenvironment can not only modulate the growth of the primary tumor but also the metastatic process [10]. Chemokines, which are soluble factors secreted by many types of cells, belong to a large superfamily and act through G-protein coupled receptors [11]. Chemokines are involved both in homeostatic functions and in inflammatory response and serve as mediators between cells [12]. Thus, the TME is involved in the inflammatory state of the tumor, which involves in particular a "chemokine storm" favoring or inhibiting tumor progression and metastasis [13,14,15,16].

We have shown earlier that CXCR2 ligand genes (CXCL1, 2, 3, 5, 6, 7, 8) are present on a narrow region of chromosome 4 in humans [17]. They contribute to the aggressiveness and chemoresistance of several types of cancers including breast [13,16,17,18,19,20,21,22]. CXCR2 ligands can be directly secreted by breast cancer cells but can also be produced by endothelial cells, mesenchymal stem cells or CAFs [15,17,20,21]. CXCR2 ligands display the Glu-Leu-Arg (ELR) motif, present in the N-terminus part of these chemokines, which is responsible for their pro-angiogenic properties [23].

CXCR2 has been shown in mouse to control a variety of functions including wound healing [24], resistance to infections [25], reproduction under microbiota influence [26], senescence [27], myelin repair [28] and metabolism [29]. The potential role of CXCR2 has been described in murine preclinical models of CXCR2 gene deletion or targeting of CXCR2 protein with antibodies. While a majority of these studies suggests that CXCR2 favors or has no effect on primary tumor growth [30,31], one study showed the contrary [32]. In human cancers, the function of CXCR2 has been scarcely investigated so far. We have shown recently that CXCR2 was mainly expressed by neutrophils in breast tumors and was present at higher levels in TNBC compared to luminal or Her2-positive breast cancers [33]. Interestingly, despite its high expression in high-grade tumors, CXCR2 predicted a lower risk of relapse [33].

The aim of this study was to use an independent, larger, cohort of TNBC patients to confirm these results and investigate whether CXCR2 levels were associated with clinicopathological variables, immune infiltration, checkpoint proteins and clinical outcome.

## 2. Materials and Methods

### 2.1. Objectives

The primary objective was to assess of the impact of CXCR2+ cell infiltration on clinical outcome of patients with TNBCs. Secondary objectives were the evaluation of the association between CXCR2+ cell infiltration and clinicopathological variables, including markers of the tumor microenvironment. 

### 2.2. Patients and Tumor Samples

The study was reviewed and approved by the Montpellier Cancer Institute Institutional Review Board (ID number ICM-CORT-2019-27). Samples were selected from a prospective collection of breast cancers surgically removed at the Institut du Cancer de Montpellier (France) between 2002 and 2010 and annotated in a dedicated prospective database (tumor biobank number BB-0033-00059). Only tumors from patients who did not have a history of another invasive cancer in the previous 5 years, without known germline BRCA mutation and with unifocal, unilateral, untreated, non-metastatic breast cancers and with ER and PR negativity (defined as <10% of tumor cells stained by IHC) [34] and HER2 negativity (defined as IHC 0/1+ or 2+ and negative fluorescent/chromogenic in situ hybridization), were selected for this study. Each tumor sample was sampled as two cores of 1mm diameter and arrayed in six tissue microarray (TMA) blocks using a manual arraying instrument (Tissue Arrayer 1, Beecher Instruments, Sun Prairie, WI, USA). 

All patients were treated according to our institutional guidelines [35] and gave their written informed consent for the use of their specimens for research purposes. As part of the study evaluating the prognostic impact of biological markers, this manuscript followed the REMARK guidelines. The clinicopathological characteristics of the study cohort are summarized in Table 1. The features of the patients enrolled in our study are consistent with the classical characteristics of TNBCs. Median age was 57.7 years (range: 28.5–89.1). Most of the tumors were T1/T2 (46.2 % and 47.6% respectively), node-negative (65.2%), ductal carcinomas (82.9%) with high histological grade (76.9%). According to our institutional guidelines [35], 75.4% of the patients received adjuvant chemotherapy. Basal-like phenotype, based on the IHC detection of CK 5/6 and/or EGFR, was observed in 64.9% of the tumors. Molecular apocrine phenotype, defined as TNBC tumors displaying both AR and FoxA1 expression, was observed in 41.8% of the studied population.

### 2.3. Immunohistochemistry

CD11b, CD66b and CXCR2 expression was analyzed by immunohistochemistry of serial sections from the same TMA blocks used in our previous studies [7,36,37,38]. For this study, we used anti-CD11b rabbit monoclonal Ab (clone EP45 at 1/400, BioSB, Santa Barbara, CA, USA), anti-CD66b mouse monoclonal (clone 80H3 at 1/200, BioRad, Marnes-la-Coquette, France) and the recently validated anti-CXCR2 mouse monoclonal antibody (clone E-2 at 1/500, Santa Cruz Technology, Dallas, TX, USA) as previously described [33]. The rabbit monoclonal SP142 antibody (Ventana Medical Systems, Tucson, AZ, USA) was used for PD-L1 detection. The detailed IHC procedures of other IHC markers used in this study (ER, PR, HER2, EGFR, CK5/6, AR, FOXA1, CD3, CD8, PD-L1 and PD1) and retrieved from other previously published studies are available in the corresponding publications [7,36,37,38].

The CD11b-, CD66b- and CXCR2-immunostained TMA sections were digitalized using a NanoZoomer slide scanner system (Hamamatsu Photonics, Hamamatsu City, Shizuoka Pref. Japan) with a 20× objective. Quantification of IHC staining was performed by image analysis using HistoLab^®^ Image Analysis Software (Microvision, Evry, France) as previously described [18] by delineating the invasive component in each sample core as a region of interest (ROI). Density of immune-reactive cells in the ROI, recorded as the number of positive cells per cm^2^, was calculated for each tumor sample and finally matched to clinicopathological data. Missing TMA cores, those with folded tissue or demonstrating significant artifacts were not scored. 

### 2.4. Evaluation of TILs 

For each sample, TIL density was assessed on corresponding HES-stained digitalized TMA sections, following the guidelines issued by the International TIL Working Group [39]. Stromal TILs were reported as the percentage of area occupied by TILs relative to the whole stroma area. 

### 2.5. Statistical Analysis

The categorical variables were described by the number of observations and the frequency of each modality. They were compared with Pearson’s chi-square test or Fisher’s exact test when the theoretical numbers were less than 5. Continuous variables were described by the median, the minimum and the maximum. OS represents the time between the date of surgery and the date of death, regardless of the cause. Details of patients alive or lost to follow-up were recorded at the last documented visit. The RFS represents the time between the date of surgery and the date of recurrence. Deaths prior to recurrence were recorded on the date of death. The most recent details for patients alive without recurrence and those lost to follow-up were also recorded. The Kaplan–Meier method was used to estimate median survival rates and times. Survival distributions were compared with the log-rank test. The hazard ratios (HRs) and their 95% confidence intervals (95% CIs) were estimated using a Cox proportional hazard model. Statistical analyses were performed with STATA 16.0 (StatCorp, College Station, TX, USA).

## 3. Results

### 3.1. Correlations of CD11b, CD66b and CXCR2 Expression with Clinicopathological Features 

In order to study the role of CXCR2 and its possible link with immune infiltration in TNBC patients, we analyzed the densities of CD11b (a marker of granulocytes), CD66b (a marker of neutrophils) and CXCR2-positive cells on a cohort of 290 samples from patients with TNBCs that we had previously characterized for both their phenotypic characteristics and their immune microenvironment [37,38]. The main characteristics of the studied population are described in Table 1 and are consistent with the classical characteristics of TNBCs (for further details, refer to Materials and Methods). This section may be divided by subheadings. It should provide a concise and precise description of the experimental results, their interpretation, as well as the experimental conclusions that can be drawn.

Densities of CD11b-, CD66b- and CXCR2-positive cells (Table 2) were not correlated to the size of the tumors, their lymph node status or basal status, but a high density of these cells was significantly associated with high-grade tumors (*p* < 0.001 for the three populations). Conversely to CD11b, which was more frequently observed in young patients (*p* = 0.018), high density of CD66b was more often observed in older patients (*p* = 0.040); no significant difference of CXCR2-positive cell density was observed according to age. When looking at the types of TNBC, high CXCR2 levels were correlated with ductal tumors (*p* = 0.041). Moreover, tumors with molecular apocrine phenotype, i.e., expressing both AR and FOXA1 biomarkers, exhibited significantly lower density of intra-tumor CD11b- or CXCR2-positive cells (*p* = 0.001 and *p* = 0.006, respectively). There was no correlation of any of the three markers with the basal-like TNBC phenotype. Finally, high density of CD11b and low density of CD66b were more frequently associated with prescription of adjuvant chemotherapy (*p* = 0.041 and *p*= 0.019, respectively).

### 3.2. Correlations between CD11b, CD66b and CXCR2 in TNBC Samples

Because we previously demonstrated that CXCR2 stained mainly stromal cells and in particular neutrophils [33], we turned our attention to the correlations between CXCR2 and CD11b and CD66b. We observed a difference in terms of tumor infiltration for CD11b-, CD66b- and CXCR2-positive cells (*p* < 0.001), with CXCR2 showing the highest infiltration (median: 1591 CXCR2+ cells/cm²; range: 0–540,653 CXCR2+ cells/cm²), followed by CD11b (median: 1305 CD11b+ cells/cm²; range: 0–205,122 CD11b+ cells/cm²) and CD66b (median: 90 CD66b+ cells/cm²; range: 0–431,006 CD66b+ cells/cm²). For each population, the median value was used as a threshold to define low or high densities.

**Figure 1:**
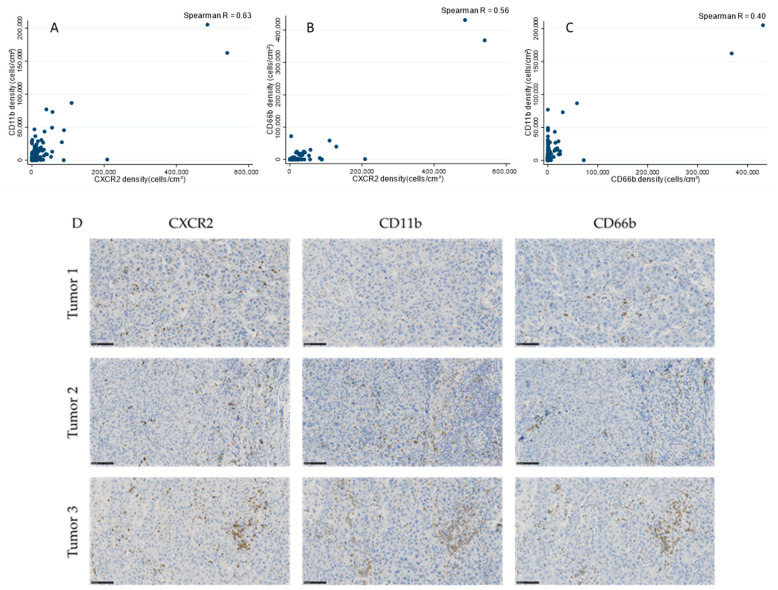
Correlations between CXCR2+ cell density and (**A**) CD11b+ cell density (Spearman’s rho = 0.63), (**B**) CD66b infiltration (Spearman’s rho = 0.56) and (**C**) between CD11b+ and CD66b+ cell density (Spearman’s rho = 0.40) in TNBC samples. (**D**) Representative images of TNBCs with variable CXCR2+ cell densities and corresponding CD11b and CD66b infiltration. Scale bar: 100 µm.

There was a good correlation between CXCR2 and CD11b (Spearman’s rho = 0.63, Figure 1A) and a modest correlation between CXCR2 and CD66b (Spearman’s rho = 0.56, Figure 1B), and between CD11b and CD66b (Spearman’s rho = 0.40, Figure 1C). 

### 3.3. Correlations of CD11b, CD66b and CXCR2 Expression with Immune Tumor Microenvironment 

To examine the relationships between CXCR2, CD11b and CD66b with immune infiltration and response of the tumor, we evaluated their correlations with the densities of tumor-infiltrating lymphocytes (TILs), CD3+ density (T lymphocytes), CD8+ density (cytotoxic T lymphocytes), programmed cell death ligand 1 (PD-L1) staining of tumor (PD-L1TC) or stromal (PD-L1Sc) cells and programmed cell death 1 staining of stroma cells (PD-1sc) (Table 3).

High densities of both CD11b+ and CXCR2+ cells were statistically positively correlated with all the studied immune markers, i.e., TIL infiltration (*p* < 0.001, for both populations); high CD3+ infiltration (*p* < 0.001, for both populations); high CD8+ infiltration (*p* = 0.006 for CD11b, and *p* < 0.001 for CXCR2); high PD-L1 expression, both on tumor cells (*p* < 0.001, for both populations) and stromal cells (*p* = 0.001 for CD11b, and *p* = 0.002 for CXCR2) and high PD-1 expression (*p* = 0.006 for CD11b, and *p* < 0.001 for CXCR2). On the other hand, high density of CD66b+ cells was positively associated with high CD8+ infiltration (*p* = 0.018), high PD-L1 expression by stromal cells (*p* < 0.001) and high PD-1 expression (*p* < 0.001).

### 3.4. Survival Analyses

We next evaluated the prognostic value of all parameters previously quantified on the survival of the patients (Table 4). The median follow-up of our cohort was 10.1 years (95% CI [9.4–10.7]). During this period, 82 patients died (5-year overall survival, OS: 80.5%, 95% CI [75.4–84.7]) and 71 had a tumor relapse (5-year relapse-free survival, RFS: 77.9%, 95% CI [72.6–82.3]), consistent with the formerly reported clinical outcome of patients with TNBCs [2].

**Figure 2:**
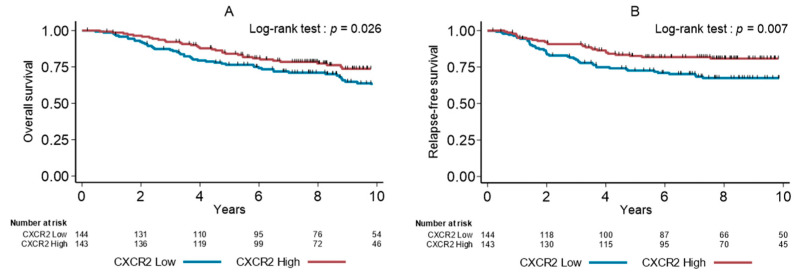
Overall survival (**A**) and relapse-free survival (**B**) in function of the tumor CXCR2+ cell density (median value used as cutoff) in the whole cohort of patients with TNBCs.

Univariate analysis (Table 4) showed that high pT and pN stages, absence of adjuvant chemotherapy, low TILs, molecular apocrine phenotype and low CXCR2+ density were significantly associated with shorter OS and RFS. The 5-year OS rates were 76.5% (95% CI [68.6–82.7]) and 84.1% (95% CI [76.8–89.2]) (*p* = 0.026), and the 5-year RFS rates were 72.6% (95% CI [64.4–79.3]) and 82.6% (95% CI [75.2–88.0]) (*p* = 0.007) in the subgroups with low and high CXCR2+ cell density, respectively (Figure 2).

OS was significantly associated with younger age (*p* = 0.001). High expression of PD-L1 by tumor cells or stromal cells was associated with longer RFS (*p* = 0.034 and *p* = 0.028, respectively). Neither CD3, CD8, CD11b nor CD66b was associated with clinical outcome.

In multivariate analysis (Table 5), tumor size, nodal involvement, ductal histology, lack of adjuvant chemotherapy and low densities of TILs or of CXCR2+ cells were independent poor prognostic factors of OS. All of these factors except histology and CXCR2 were also associated with a shorter RFS.

In addition, 14 cases in our population displayed a weak HR expression, in the 1% to 9% positivity range. We performed a separate multivariate analysis excluding these 14 cases (Table S1). The results show that high levels of CXCR2 continue to be significantly associated with a better OS (HR 0.6; 95% CI 0.38–0.97, *p* = 0.033). In addition, in this second analysis, high levels of CXCR2 were significantly associated with a better RFS (HR 0.58; 95% CI 0.34–0.97, *p* = 0.036), suggesting a greater impact of CXCR2 on clinical parameters in the subpopulation displaying no HR expression (Table S1).

**Figure 3:**
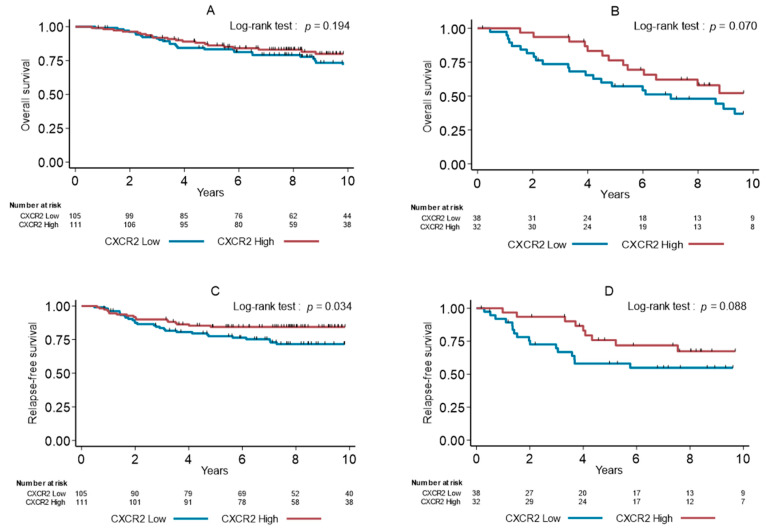
Overall survival (**A**,**B**) and relapse-free survival (**C**,**D**) in function of the CXCR2+ cell density (median value used as cutoff) in patients with TNBCs treated (**A**,**B**) or untreated (**C**,**D**) with adjuvant chemotherapy.

To evaluate the specific predictive or prognostic values of CXCR2+ cell infiltration, we analyzed OS and RFS in patients with TNBCs treated or not with adjuvant chemotherapy, respectively. In patients who had received chemotherapy, there was no effect of high density of CXCR2+ cells on OS (*p* = 0.194, Figure 3A), but CXCR2 density was significantly associated with longer RFS (*p* = 0.034, Figure 3B). In untreated patients (*n* = 70), we observed a trend between low CXCR2+ cell density and shorter OS (*p* = 0.070, Figure 3C) and RFS (*p* = 0.088, Figure 3D).

## 4. Discussion

The aim of this study was to focus on the potential roles of CXCR2, CD11b and CD66b in TNBCs, with a particular attention to immune cell infiltration and immune checkpoint markers. We first observed that high levels of the three markers were correlated to high-grade TNBCs, confirming our previous study on a smaller cohort of breast cancer patients [33]. CXCR2 stained immune granulocytic cells and not cancer cells. This includes in particular neutrophils but possibly other types of cells as well, as there was a better correlation of CXCR2 with CD11b than with CD66b. Interestingly, low expression of CXCR2 and CD11b was also associated with molecular apocrine-like TNBCs, expressing both AR and FoxA1, which represent a subgroup of TNBCs with a worse prognosis characterized by late relapses [7].

TNBCs are considered as an immunogenic breast cancer subtype and show a higher degree of infiltration by lymphocytes compared to luminal breast tumors [41], even though this infiltration tends to be lower in more advanced stages of the disease compared to early stages [42]. This could be the result of their higher production of multiple cytokines [16,17,19] or their genomic instability leading to high rates of mutations and to the production of neoantigens, which in turn increase their immunogenicity [43]. We observed that high levels of CXCR2 and CD11b were correlated with higher densities of TILs, CD3+ and CD8+ cytotoxic lymphocytes. In TNBCs, high TIL levels are generally correlated with a better prognosis [44]. In early TNBC patients treated with anthracycline-based adjuvant chemotherapy, the levels of TILs are positively correlated with a better outcome [45]. Similar results were obtained for untreated early TNBCs, showing that higher TIL densities correlated with better survival [46,47]. Similarly, high quantities of cytotoxic CD8+ TILs are positively associated with a longer survival of early TNBC patients [48] and better response to neoadjuvant chemotherapy [49]. Moreover, high levels of total T cells (based on CD3 expression) correlate with a better response to adjuvant chemotherapy of early breast cancer patients [50]. It is important to point out that more advanced stages of breast cancers display reduced immune infiltration [42]. These TILs may lose some of their immunosuppressive properties [51].

We next analyzed the presence of PD-L1- and PD-1-positive cells in our cohort. Immune checkpoints are upregulated to inhibit the cytotoxic response and prevent an excessive immune reaction [52]. PD-1 is normally expressed at the surface of T cells, B cells, natural killer cells and myeloid cells [53] whereas PD-L1 can be expressed both by tumor cells and cells from the TME, including activated T-cells, macrophages [54] and CAFs [55]. We report that high levels of CXCR2 and CD11b were correlated to higher levels of PD-L1 expression on both tumor and stromal cells as well PD-1 by stromal cells. CD66b was only correlated to PD-L1 and PD-1 expression by stromal cells. The correlation of CXCR2 levels with immune checkpoint markers might represent an attempt of the body to counteract tumor progression.

We have also analyzed the prognostic value of CXCR2 in our cohort. In univariate analysis, there was a correlation between poor OS and RFS and low levels of CXCR2. Multivariate analysis confirmed that low levels of CXCR2 were associated with poor OS. Moreover, when focusing on TNBCs treated with adjuvant chemotherapy, we observed that CXCR2 density was associated with longer RFS. A potential pitfall of our study could be linked to the choice of a European definition of TNBCs, considering a <10% negativity threshold for the determination of the hormone receptor status. To circumvent this potential issue, we analyzed our cohort using the ASCO/CAP threshold of 0 to define HR negativity [34,56] and found a greater impact of high levels of CXCR2 expression on survival parameters. This is in accordance with published evidence showing that these two groups of tumors are closely related in term of clinical behavior and mutational spectrum [57]. All this suggests that high expression of CXCR2 could favor a better outcome of TNBCs, which is also in agreement with our previous results obtained for all types of breast cancers, not just TNBCs [33]. This is consistent with the fact that CXCR2 is expressed in fewer cells in molecular apocrine-like tumors, which have a worse long term prognosis than other subtypes of TNBCs, and that CXCR2 levels correlate with TILs, CD8+ and CD3+, which are associated with a better prognosis. On the other hand, the presence of a high number of CXCR2 cells in tumors characterized by high expression of PD-L1 on both tumor and stromal cells, but also in tumors exhibiting high level of PD-1, could represent an attempt of the immune cells to counteract the immune escape that is promoted by the PD1/PD-L1 pathway.

## 5. Conclusions

In TNBCs, the expression of the chemokine receptor CXCR2 is associated with higher immune infiltration and a more favorable outcome, despite its correlation with the presence of PD-L1TC, PD-L1sc or PD-1sc cells. Its expression varies between TNBC subtypes, with a lower expression rate in molecular apocrine-like tumors, a subtype of TNBC characterized by later recurrence risk and worse prognosis. Whether these results indicate a direct role of CXCR2 in the control of the effects of immune checkpoint proteins, such as PD-L1 and PD-1, will require further investigation.

## Figures and Tables

**Table 1:** Clinicopathological characteristics of the studied population (*n* = 290).

Patient Features		Number of Patients(*n* = 290)	%
Age (years), median (min to max)		57.72	[28.54–89.10]
	<55	129	44.5
	≥55	161	55.5
Tumor size			
	T1	134	46.2
	T2	138	47.6
	T3/T4	18	6.2
Nodal status			
	N−	189	65.2
	N+	101	34.8
Histological grade (4 missing values)			
	1–2	66	23.1
	3	220	76.9
Histology (3 missing values)			
	Ductal	238	82.9
	Lobular	15	5.2
	Other ^(1)^	34	11.9
Adjuvant chemotherapy (1 missing value)			
	No	71	24.6
	Yes	218	75.4
Basal-like phenotype (2 missing value)			
	No	101	35.1
	Yes (basal)	187	64.9
Molecular apocrine phenotype (17 missing values)			
	No	159	58.2
	Yes (molecular apocrine)	114	41.8
TIL %, median (6 missing values)			
	<5%	134	47.2
	≥5%	150	52.8
CD3+ cell density (2 missing values)			
	Low	144	50.0
	High	144	50.0
CD8+ cell density (6 missing values)			
	Low	142	50.0
	High	142	50.0
PD-L1_TC_ (24 missing values)			
	<1%	119	44.7
	≥1%	147	55.3
PD-L1_SC_ (27 missing values)			
	0	48	18.3
	[0–10]	85	32.3
	[10–50]	72	27.4
	≥50	58	22.1
PD-1_SC_ (21 missing values)			
	0	69	25.7
	[0–10]	72	26.8
	[10–50]	106	39.4
	≥50	22	8.2
CD11b+ cell density (15 missing values)			
	Low	137	49.8
	High	138	50.2
CD66b+ cell density (14 missing values)			
	Low	138	50.0
	High	138	50.0
CXCR2+ cell density (3 missing values)			
	Low	144	50.2
	High	143	49.8

Footnote: Basal-like phenotype was considered in the case of positive staining for cytokeratin 5/6 and/or EGFR (>10% of tumor cells stained in IHC); Molecular apocrine phenotype was defined in TNBC tumors that express both androgen receptor (AR) and Forkhead box protein A1 (FOXA1) biomarkers; TILs: tumor-infiltrating lymphocytes according to Salgado guidelines [40]; PD-L1: programmed cell death ligand 1; PD-1: programmed cell death 1; TC: tumor cells; SC: stromal cells. ^(1)^ Seven metaplastic, seven invasive papillary, seven medullary, six mixed invasive, two apocrine, one adenoid cystic, one neuroendocrine, one mucinous, one polymorphous carcinoma and one malignant myoepithelioma.

**Table 2:** Correlations of CD11b, CD66b and CXCR2 expression with clinicopathological features.

Variables		CD11b					CD66b					CXCR2				
		Low		High		*p*-Value	Low		High		*p*-Value	Low		High		*p*-Value
		N	%	N	%		N	%	N	%		N	%	N	%	
Age (years)																
	<55	53	38.7	73	52.9	0.018	72	52.2	55	39.9	0.040	61	42.4	68	47.6	0.377
	≥55	84	61.3	65	47.1		66	47.8	83	60.1		83	57.6	75	52.5	
Tumor size																
	T1	56	40.9	71	51.5	0.079	70	50.7	57	41.3	0.116	68	47.2	64	44.8	0.675
	T2/T3/T4	81	59.1	67	48.6		68	49.3	81	58.7		76	52.8	79	55.2	
Nodal status																
	N−	84	61.3	95	68.8	0.190	88	63.8	92	66.7	0.613	90	62.5	97	67.8	0.343
	N+	53	38.7	43	31.2		50	36.2	46	33.3		54	37.5	46	32.2	
Histological grade																
	1–2	48	35.8	14	10.2	<0.001	47	34.8	14	10.2	<0.001	49	34.8	16	11.3	<0.001
	3	86	64.2	123	89.8		88	65.2	123	89.8		92	65.3	126	88.7	
Histology																
	Ductal	109	79.6	118	87.4	0.082	109	79.6	120	88.2	0.051	111	78.2	124	87.3	0.041
	Other	28	20.4	17	12.6		28	20.4	16	11.8		31	21.8	18	12.7	
Adjuvant chemotherapy																
	No	40	29.4	26	18.8	0.041	25	18.3	42	30.4	0.019	38	26.6	32	22.4	0.409
	Yes	96	70.6	112	81.2		112	81.8	96	69.6		105	73.4	111	77.6	
Basal-like phenotype																
	No	55	40.4	41	29.9	0.069	46	33.8	52	37.7	0.505	56	39.4	45	31.5	0.160
	Yes (Basal)	81	59.6	96	70.1		90	66.2	86	62.3		86	60.6	98	68.5	
Molecular apocrine phenotype																
	No	61	48.4	91	68.4	0.001	71	55.9	83	61.5	0.359	66	50.0	93	66.4	0.006
	Yes (Molecular apocrine)	65	51.6	42	31.6		56	44.1	52	38.5		66	50.0	47	33.6	

Footnote: Basal-like phenotype was considered in the case of positive staining for cytokeratin 5/6 and/or EGFR (>10% of tumor cells stained in IHC); Molecular apocrine phenotype was defined in TNBC tumors that express both AR (androgen receptor) and FOXA1 (Forkhead box protein A1) biomarkers.

**Table 3:** Correlations of CD11b, CD66b and CXCR2 expression with immune tumor microenvironment.

Variables		CD11b Density					CD66b Density					CXCR2 Density				
		Low		High		*p*-Value	Low		High		*p*-Value	Low		High		*p*-Value
		N	%	N	%		N	%	N	%		N	%	N	%	
TILs																
	<5%	88	64.7	39	29.1	<0.001	70	51.1	56	41.8	0.125	84	60.0	49	34.8	<0.001
	≥5%	48	35.3	95	70.9		67	48.9	78	58.2		56	40.0	92	65.3	
CD3+ density																
	Low	94	69.1	42	30.4	<0.001	73	52.9	63	45.7	0.229	91	63.6	52	36.4	<0.001
	High	42	30.9	96	69.6		65	47.1	75	54.4		52	36.4	91	63.6	
CD8+ density																
	Low	78	58.2	57	41.6	0.006	77	56.2	57	41.9	0.018	90	63.8	51	36.2	<0.001
	High	56	41.8	80	58.4		60	43.8	79	58.1		51	36.2	90	63.8	
PD-L1_TC_																
	<1%	68	56.7	45	33.8	<0.001	59	48.0	52	39.4	0.168	72	56.7	47	34.1	<0.001
	≥1%	52	43.3	88	66.2		64	52.0	80	60.6		55	43.3	91	65.9	
PD-L1_SC_																
	<10%	72	61.0	53	39.9	0.001	77	62.6	49	37.7	<0.001	76	60.8	57	41.6	0.002
	≥10%	46	39.0	80	60.2		46	37.4	81	62.3		49	39.2	80	58.4	
PD-1_SC_																
	<10%	76	61.8	59	44.7	0.006	84	65.6	51	38.9	<0.001	84	63.6	57	41.9	<0.001
	≥10%	47	38.2	73	55.3		44	34.4	80	61.1		48	36.4	79	58.1	

Footnote: TILs: tumor-infiltrating lymphocytes according to Salgado guidelines [40]; PD-L1: programmed cell death ligand 1; PD-1: programmed cell death 1; TC: tumor cells; SC: stromal cells.

**Table 4:** Univariate analysis of survivals.

Variable		OS			RFS		
		HR	95% CI	*p*-Value	HR	95% CI	*p*-Value
Age (years)							
	<55	1			1		
	≥55	2.07	1.31–3.27	0.001	1.43	0.89–2.31	0.137
Tumor size							
	T1	1			1		
	T2/T3/T4	2.82	1.75–4.55	<0.001	2.59	1.55–4.34	<0.001
Nodal status							
	N−	1			1		
	N+	2.25	1.48–3.42	<0.001	4.34	2.67–7.05	<0.001
Histological grade							
	1–2	1			1		
	3	0.79	0.50–1.26	0.328	1.02	0.59–1.76	0.931
Histology							
	Ductal	1			1		
	Other	0.61	0.33–1.15	0.108	0.91	0.49–1.69	0.764
Adjuvant chemotherapy							
	No	1			1		
	Yes	0.33	0.21–0.50	<0.001	0.5	0.31–0.81	0.007
Basal-like phenotype							
	No	1			1		
	Yes (basal)	1.06	0.68–1.66	0.787	0.85	0.53–1.36	0.495
Molecular apocrine phenotype							
	No	1			1		
	Yes (molecular apocrine)	1.6	1.04–2.46	0.033	1.65	1.03–2.63	0.038
TILs							
	<5%	1			1		
	≥5%	0.52	0.33–0.80	0.003	0.47	0.29–0.76	0.002
CD3+ density							
	Low	1			1		
	High	0.72	0.47–1.10	0.126	0.64	0.40–1.02	0.059
CD8+ density							
	Low	1			1		
	High	1.11	0.72–1.70	0.634	0.91	0.57–1.45	0.696
PD-L1_TC_							
	<1%	1			1		
	≥1%	0.66	0.42–1.02	0.061	0.59	0.37–0.96	0.034
PD-L1_SC_							
	<10%	1			1		
	≥10%	0.67	0.42–1.06	0.081	0.57	0.35–0.95	0.028
PD-1_SC_							
	<10%	1			1		
	≥10%	1.09	0.71–1.67	0.708	0.92	0.57–1.47	0.725
CD11b density							
	Low	1			1		
	High	0.72	0.46–1.12	0.141	0.66	0.40–1.07	0.088
CD66b density							
	Low	1			1		
	High	1.29	0.83–2.01	0.251	1.2	0.74–1.93	0.456
CXCR2 density							
	Low	1			1		
	High	0.61	0.40–0.95	0.026	0.52	0.32–0.85	0.007

Footnote: OS: overall survival; RFS: relapse-free survival; HR: hazard ratio; CI: confidence interval; Basal-like phenotype was considered in the case of positive staining for cytokeratin 5/6 and/or EGFR (>10% of tumor cells stained in IHC); Molecular apocrine phenotype was defined in TNBC tumors that express both androgen receptor (AR) and Forkhead box protein A1 (FOXA1) biomarkers; TILs: tumor-infiltrating lymphocytes; PD-L1: programmed cell death ligand 1; PD-1: programmed cell death 1; TC: tumor cells; SC: stromal cells.

**Table 5:** Multivariate analysis of survivals.

Variables		OS			RFS		
		HR	95% CI	*p*-Value	HR	95% CI	*p*-Value
Tumor size				<0.001			0.017
	T1	1			1		
	T2/T3/T4	2.48	1.49–4.13		1.87	1.10–3.17	
Nodal status				<0.001			<0.001
	N−	1			1		
	N+	2.51	1.59–3.97		4.28	2.57–7.12	
Adjuvant chemotherapy				<0.001			0.002
	No	1			1		
	Yes	0.32	0.20–0.50		0.43	0.26–0.71	
Histology				0.002			
	Ductal	1					
	Other	0.38	0.19–0.76				
TILs				0.008			0.01
	<5%	1			1		
	≥5%	0.54	0.34–0.86		0.52	0.31–0.86	
CXCR2				0.05			0.058
	Low	1			1		
	High	0.64	0.40–1.01		0.61	0.37–1.02	

Footnote: OS: overall survival; RFS: relapse-free survival; HR: hazard ratio; CI: confidence interval; TILs: tumor-infiltrating lymphocytes.

## Data Availability

The data presented in this study are available on request from the corresponding author.

## Supplementary Materials

The following are available online at https://www.mdpi.com/article/10.3390/cancers13102328/s1, Table S1: Multivariate analysis of survivals with exclusion of the 14 patients with ER and/or PR expression between1 and 9%.

## Abbreviations

AB	Antibody
AR	Androgen receptor
CAFs	Cancer associated fibroblasts
CI	Confidence interval
ECM	Extracellular matrix
ER	Estrogen receptor
FOXA1	Forkhead box protein A1
HR	Hazard ratio
IHC	Immunohistochemistry
NK	Natural killer
OS	Overall survival
PR	Progesterone receptor
ROI	Region of interest
RFS	Relapse-free survival
TIL	Tumor-infiltrating lymphocytes
TMA	Tissue microarray
TME	Tumor microenvironment
TNBC	Triple-negative breast cancer
