# Supplementary material for: CXCR2 Levels Correlate with Immune Infiltration and a Better Prognosis of Triple-Negative Breast Cancers
Source: Cancers (Basel). 2021 May 12;13(10):2328. doi: 10.3390/cancers13102328 (PMC8151934; doi:10.3390/cancers13102328)
Supplement: Supplementary file 1 [file cancers-13-02328-s001.zip › cancers-1196179-supplementary.pdf]

Variables	OS			RFS		
	HR	95% CI	P-value	HR	95% CI	P-value
**Tumor size**						
T1	1		<0.001	1		0.016
T2/T3/T4	2.53	1.50 - 4.26		1.90	1.11 - 3.27	
**Nodal status**						
N-	1		<0.001	1		<0.001
N+	2.54	1.59 - 4.05		4.27	2.54 - 7.17	
**Adjuvant chemotherapy**						
No	1		<0.001	1		0.009
Yes	0.36	0.22 - 0.57		0.48	0.28 - 0.81	
**Histology**						
Ductal	1		0.003			
Other	0.38	0.19 - 0.77				
**TILs**						
< 5	1		0.012	1		0.021
≥ 5	0.55	0.34 - 0.88		0.55	0.33 - 0.92	
**CXCR2**						
Low	1		0.033	1		0.036
High	0.60	0.38 - 0.97		0.58	0.34 - 0.97	

Supplemental Table 1: Multivariate analysis of survivals with exclusion of the 14 patients with ER and/or PR expression between1 and 9%
